# Do Bird Assemblages Predict Susceptibility by E-Waste Pollution? A Comparative Study Based on Species- and Guild-Dependent Responses in China Agroecosystems

**DOI:** 10.1371/journal.pone.0122264

**Published:** 2015-03-26

**Authors:** Qiang Zhang, Jiangping Wu, Yuxin Sun, Min Zhang, Bixian Mai, Ling Mo, Tien Ming Lee, Fasheng Zou

**Affiliations:** 1 Guangdong Entomological Institute/South China Institute of Endangered Animals, Guangzhou, China; 2 Guangzhou Institute of Geochemistry, Chinese Academy of Sciences, Guangzhou, China; 3 Department of Ecology, Evolution, and Environmental Biology and Earth Institute, Columbia University, New York, New York, United States of America; Universidad de Granada, SPAIN

## Abstract

Indirect effects of electronic waste (e-waste) have been proposed as a causal factor in the decline of bird populations, but analyses of the severity impacts on community assembly are currently lacking. To explore how population abundance/species diversity are influenced, and which functional traits are important in determining e-waste susceptibility, here we surveyed breeding and overwintering birds with a hierarchically nested sampling design, and used linear mixed models to analyze changes in bird assemblages along an exposure gradient in South China. Total bird abundance and species diversity decreased with e-waste severity (exposed < surrounding < reference), reflecting the decreasing discharge and consequent side effects. Twenty-five breeding species exclusively used natural farmland, and nine species decreased significantly in relative abundance at e-waste polluted sites. A high pairwise similarity between exposed and surrounding sites indicates a diffuse effect of pollutants on the species assembly at local scale. We show that sensitivity to e-waste severity varies substantially across functional guild, with the prevalence of woodland insectivorous and grassland specialists declining, while some open farmland generalists such as arboreal frugivores, and terrestrial granivores were also rare. By contrast, the response of waterbirds, omnivorous and non-breeding visitors seem to be tolerable to a wide range of pollution so far. These findings underscore that improper e-waste dismantling results in a severe decline of bird diversity, and the different bird assemblages on polluted and natural farmlands imply species- and guild-dependent susceptibility with functional traits. Moreover, a better understanding of the impact of e-waste with different pollution levels, combined multiple pollutants, and in a food-web context on bird is required in future.

## Introduction

Bird species have shown substantial population decline and range contraction in agroecosystems, which have been linked to the intensification of agriculture [1–4]. Potential mechanisms, including the effects of pollutants, are reviewed by Fuller [5] and Newton [6]. Several studies have explored how regular pollutants detrimentally affect bird populations. For instance, the increased use of pesticides and inorganic fertilizers depresses food supply [6–8], while point sources of non-ferrous smelters pose a wide-ranging hazard for breeding performance and local survival of birds [9,10]. Although the toxicology effects of pollutants are now more evident than before (e.g., individual levels for target species), evidence for the potential side effects that alters community structure with emerging pollutants, and the causal links of species-specific and functional-guild biotic responses remains scarce.

Electronic waste (e-waste) has draw world’s attention specifically to the indiscriminate toxicity of persistent organic pollutants (POPs), particularly in developing countries [11, 12]. As the world’s largest importing and recycling center, China has been faced with severe e-waste contamination since the early 1990s [13]. Due to primitive cabin dismantling (e.g., open burning, acid leaching, toner sweeping), large quantities of POPs, such as polychlorinated biphenyls (PCBs) and polybrominated diphenyl ethers (PBDEs) are released into surrounding farmland. Recent study has shown that the average concentrations of these pollutants at or near the site are 1 or 2 orders of magnitude higher than those of background farmland soils or reservoir sediments [14]. It has also been accumulated in fish, frogs, birds and even human tissues collected from several hot spots (e.g., Guiyu, Longtang and Shijiao), thus providing data for an assessment of the emerging e-waste pollution issue [15–20]. Since the substantial variability in contamination levels of biota may be mediated by the characteristics of habitat preferences, dietary specialization, and migratory status [21], it is essential to understand the ecological consequences of contamination in community level, and compare them with reference sites.

Functionally, birds are one of the most diverse groups of vertebrate, and evidences indicate that bird functional-guilds respond differently to habitat changes [22,23]. Farmland birds have diverse habitat preference and trophic level compared with forest species, so it is susceptible to e-waste pollution. Notably, several POPs have been shown to disrupt thyroid hormones, affect liver and kidney morphology, neurobehavioural development and reproductive function, and have fetal toxicity/teratogenicity in lab animals and wildlife [24]. Based on our earlier toxicology studies, different POPs have been accumulated and biomagnified in several passerines and waterbirds [17, 25], and the level of PBDEs is dependent on bird dietary or trophic position (carnivore > insectivore > frugivore) [26–28]. Now that e-waste pollution persisted for more than three decades in South China, it is urgent to know how population abundance and species diversity of farmland birds changed compared with reference sites, and which species-specific or guild-dependent traits are functionally important in determining the susceptibility to e-waste pollution.

Here we examine species and guild responses of farmland bird communities to the effects of e-waste pollution. By using a nested sampling design, we measured bird population abundance and species diversity from e-waste exposed, surrounding, and natural reference sites in the Pearl River Delta, China. Specifically, the objectives of this study are threefold: (1) to compare relative abundance, species diversity, and community composition of birds between e-waste polluted and natural farmlands, with the latter expected to present a higher diversity level; (2) to investigate which species or functional groups (different taxa or guilds) are susceptible to e-waste pollution, and which are resistant in community level; (3) to identify challenges for future conservation effort of farmland bird species at e-waste regions in South China.

## Methods

### Ethics statement

No specific permissions were required for the use of bird point count locations as they occurred on public right of ways (e.g., roadside, farmland and countryside). As private farmland owners in e-waste recycling regions did not want their information posted publically please contact the authors for contact details. The field studies did not involve endangered or protected species. This study did not require approval from an Animal Care and Use Committee because it was a non-invasive observational field study, and did not involve the capture and handling of wild animals.

### Study areas

**Fig 1:**
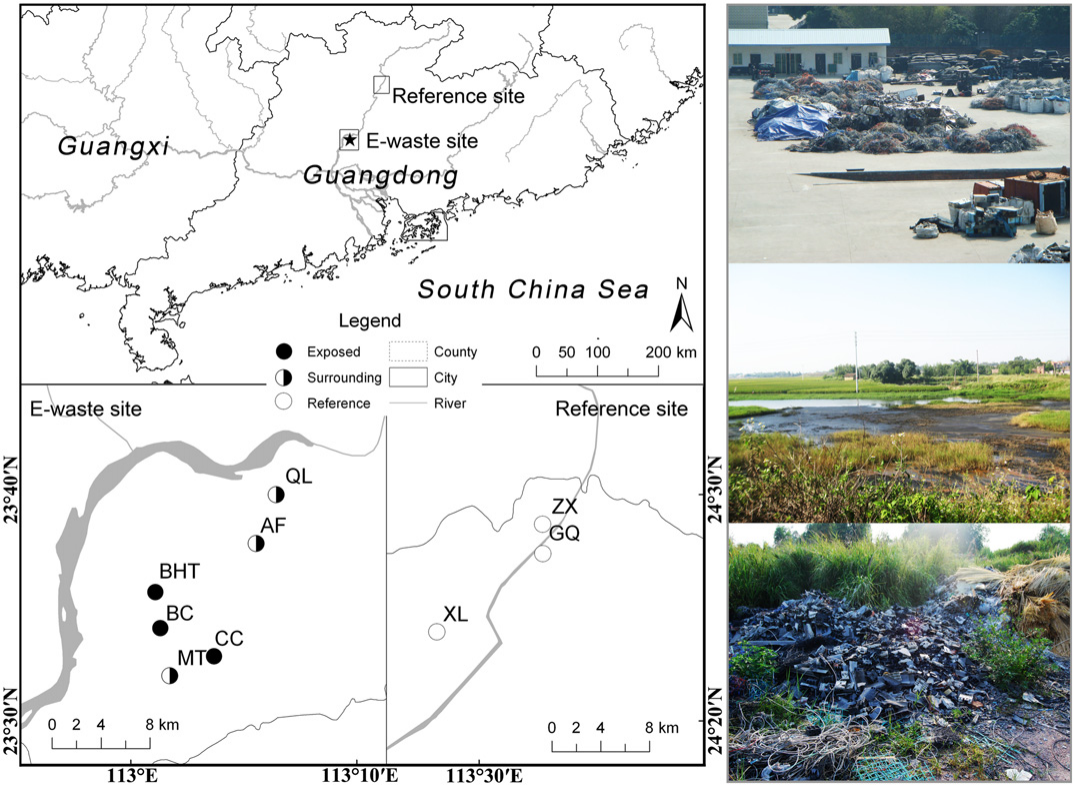
Location of the e-waste exposed, e-waste surrounding and natural reference sites where bird population densities were measured in Guangdong, China. Study sites are coded by different treatments as black circles (exposed, i.e. BHT-Baihetang, CC-Changchong, BC-Banchong), white and black circles (surrounding, i.e. MT-Matou, QL-Qinglian, AF-Anfeng), and white circles (natural, i.e. ZX-Zhouxi, XL-Xinliang, GQ-Gaoqiao). Photo tip: the scene of recycling workshop, polluted river and dumping site in e-waste area.

The data were collected at e-waste exposed, surrounding and reference zone in South China (Fig. 1). The e-waste exposed sites are in Qingyuan, Guangdong Province (one of China's main manufacturing zones—the Pearl River Delta/PRD), which has also been a major hub for the disposal of e-waste in China. These sites hold more than 1300 dismantling and recycling workshops, and about 1.7 million tons of e-waste are dismantled and recycled annually by over 80,000 workers [17]. In recycling areas (113°01′–113°06′E, 23°32′–23°41′N, in the town of Longtang), e-waste is disposed of to recover the metal and other useful components using primitive techniques, such as combusting or roasting the circuit boards on open fires in workshops, extracting metals using strong acids (with resultant dumping of acidic wastes), and open burning of cables and e-waste. All these inappropriate recycling activities release a large amount of toxic chemicals into the immediate environment, which have adverse health effects on humans and wildlife [14]. Surrounding areas were also measured, since biota may be influenced by adjacent contamination, particularly for non-point source pollution. For comparison, we collected samples as reference groups from the town of Shakou, Yingde (113°27′–113°33′E, 24°23′–24°29′N, about 120 km away from the e-waste exposed areas), which lies in the upstream of the Beijiang River and contiguous to Shimentai Nature Reserve, and therefore its remoteness from industry indicates that the influence of POPs is negligible [29].

Because of the mosaic of agricultural systems in the study area, each transect crossed plots with different types of soil cover, such as arable, fallows, grasses, woods, water-areas, and buildings. All sampling sites were characterized by a mixture of agroecosystems in the elevational range of 100–300 m asl. To reduce possible confounding bias due to sampling with different habitat heterogeneity, we first selected transects randomly in landscape, and guaranteed that there was no significant difference among severity groups for each variables (Table 1, Upper part, all *p* values are greater than 0.05). For all points used in analysis, farmland comprised ≥ 55% of the transect cover. In addition, based on our earlier studies, **i**nformation on the types and levels of e-waste-related chemical POPs concentrations in birds were also listed at Table 1 (Lower part).

**Table 1:** Upper part: the major habitat characteristics where birds were censused for this study.

Habitat characteristics (%)	Exposed	Surrounding	Reference
Farmland cover	57.7 ± 8.4^a^	65.4 ± 12.8^a^	57.0 ± 2.4^a^
Woodland cover	13.4 ± 4.6^a^	13.3 ± 9.7^a^	23.2 ± 7.3^a^
Grassland cover	3.9 ± 0.9^a^	5.6 ± 1.0^a^	5.1 ± 0.2^a^
Water-area cover	8.9 ± 6.4^a^	9.37 ± 4.8^a^	4.6 ± 3.5^a^
Building cover	16.1 ± 9.4^a^	6.2 ± 6.2^a^	10.1 ± 4.4^a^
**E-waste POPs concentrations**	**Exposed**	**Surrounding**	**Reference**
**∑PCBs**			
Common Kingfisher	26800 (21360–1526500)^a^	-	320(149–1064)^a^
Chinese Bulbul	6600 (3200–73000)^c^	-	108 (45–183)^a^
Magpie Robin	48000 (6100–190000)^a^	-	240 (72–380)^a^
**∑PBDEs**			
Common Kingfisher	8760 (2030–26400)^e^	-	87(25–384)^a^
Chinese Bulbul	1000 (630–4700)^b^	-	27 (12–45)^a^
Magpie Robin	5200 (870–15000)^b^	-	81 (29–340)^a^
**DP**			
Common Kingfisher	58 (29–150)^d^	-	2.97 (0.9–24)^a^
Chinese Bulbul	21 (12–46)^b^	-	4.1 (2.0–15.0)^a^
Magpie Robin	110 (39–930)^a^	-	3.9 (1.2–16.6)^a^
**DBDPE**			
Common Kingfisher	12 (4.5–52)^c^	-	3.9 (1.2–24)^a^
Chinese Bulbul	33 (27–60)^b^	-	11 (4.5–19)^a^
Magpie Robin	21 (12–46)^b^	-	11 (2.7–82)^a^

Lower part: Information on the types and levels of e-waste-related chemical POPs concentrations (median and range, ng/g lipid weight) in birds.

*Notes of upperpart*: Within rows, same susuperscripts indicate that no siginificant difference are based on are based on one-way ANOVA (df = 2, 8) with Tukey’s test for multiple comparisons

*Source of lower part*:

^a^ Unpublished data (in press);

^b^ Sun et al. (2012) [27];

^c^ Sun et al. (2014) [28];

^d^ Mo et al. (2013) [29];

^e^ Mo et al. (2012) [30].

### Bird surveys

Farmland birds have been commonly defined in Europe as those found primarily in farmed open habitats [6]. Given the unique agroecosystem in South China (with a mix of paddy, woods, grasses, water-areas and buildings), we adopted a broader view of farmland birds that includes almost all land birds that breed and overwinter in open habitats. Bird surveys were carried out consecutively for two winters (December 2011 and January 2012) and two breeding seasons (June 2011 and July 2012), and a nested sampling design was used to establish near-complete inventories of bird assemblages. Nine transects were established, three in each sampling area, with each transect consisting of 10 points at least 200 m from each other. Point counts were used to assess abundance of bird species, noting all individuals recorded within the limit of the experimental replicate during the time of census. The censuses began at sunrise and ended before 10:00 a.m. on windless and rainless days. Within a 50 m radius plot, two observers simultaneously recorded all birds either by visual or auditory detection lasting 10 min. A digital rangefinder was used to measure and estimate distances, and all observations beyond 50 m were discarded for analyses of site species richness. The sampling plots were geo-referenced with a portable GPS, and covered all main habitats (i.e. farmland, woodland, wetland and farmer-settlements). Almost all transects consisted of a mixture of these habitats, and the data were a representative sample with homogeneous landscape among e-waste exposed, surrounding and reference units.

### Functional traits

Based on the datasets of Zhao (2001) and Zhang et al. (2011) [31–32], with updates from field experience, we classified species according to three key ecological or functional traits: habitat preference, dietary guild and migratory status. A guild-by-site matrix was constructed by counting species in each guild at each site. according to their primary use of habitat type and structure for nesting and movement, species were first categorized into one of nine habitat preference: “artificial marshland or wetland (AW)”; “aquatic ponds and paddy (AP)”; “woodland specialist (WS)”; “edge-tolerant woodland species (EWS)”; “non-forest dependent species (e.g., plantation and orchard; NFS)”; “generalist (G)”; “aerial species (A)”; “grassland and shrub users (GSU)” and “open-habitat species (OS)”. Species were then allocated one of 11 dietary guilds: “carnivore (CA)”; “arboreal foliage glean insectivore (AI)”; “arboreal foliage glean insectivore-frugivore (AIF)”; “sallying insectivore (SI)”; “terrestrial insectivore (TI)”, “miscellaneous insectivore (MI)”; “terrestrial insectivore-frugivore (TIF)”; “arboreal frugivore (AF)”; “terrestrial granivore (TG)”; “miscellaneous insectivore-piscivore (MIP)” and “aquatic invertebrate (AQI)”. Species were also assigned to one of four categories of migrations status: permanent resident (R), winter visitor (W), summer visitor (S), and passage migrant (P). For a complete list of species with functional traits and data, see Supporting Information (S1 Table).

### Statistical analysis

To compare bird assemblages among the e-waste exposed, surrounding and reference regions, we first assessed whether our sampling effort was sufficient to represent the species richness of each region sampled using Ecosim 7.0 [33]. Then, mean point species richness, abundance and diversity among different sites were calculated using PC-ORD 5.0 [34]. Species richness was considered as the mean number of species appearing at each point throughout the four censuses. For relative abundance, the response variable was the average number of individuals found per sampling point. Diversity was estimated using the Shannon diversity index (*H’* = Σ*Pi* × LnP_*i*_, where *P*_*i*_ is the relative abundance of species *i*). For habitat characteristics, the differences among three regions were tested with a one-way ANOVA, followed by a post hoc Tukey test (SPSS, 17.0).

At the transect level, we used a linear mixed model (LMM) to highlight the e-waste severity that affect overall bird density, Shannon diversity, and the abundance of the three functional groups while simultaneously controlling for potential spatial non-dependence of transects [with sampling point nested in zone (contaminated vs. reference) as random factor]. In our case, primary sample units consisted of transects, each made up of 10 secondary sample units (points). Because we did not control what the e-waste severity was, or whether severity was homogeneous among points within a transect. The advantage of LMM framework allows for inferences to be applied to the entire population from which samples were draw [35], by treating ‘transect point’ as a random effect, while e-waste severity as the fixed factor (exposed, surrounding and reference). All LMM analyses were carried out using SPSS 19. These parameters were estimated using the maximum likelihood method, and the test was carried out using a type III sums-of-squares *F* test.

To examine relationships among the nine sites, we used Sorensen (qulitative) index scores (beta diversity) as inputs into a cluster analysis using average linkage clustering to group sites by community composition following PC-ORD 5.0. Then gradient analysis method was used to summaries information on bird assemblage among different regions in CANOCO 4.5 [36]. A preliminary detrended correspondence analysis (DCA) showed a maximum gradient length of 5.681 (detrending by segments), which suggested a unimodal distribution of data. Subsequently, correspondence analysis (CA) was performed to detect the correlative patterns between sampling points and bird assemblage, with the original dissimilarities scores as input. Because the proportions of nine habitat-user guilds are inter-correlated and do not vary independently, to indicate which particular treatment influenced the habitat-user guilds, we performed nonparameter tests based on 1000 permutations to select significantly different habitats between groups at an alpha level of *p* < 0.05 and illustrate the significant habitats as vectors on the CA ordination diagram.

## Results

### Population abundance and species diversity (spot diversity)

**Fig 2:**
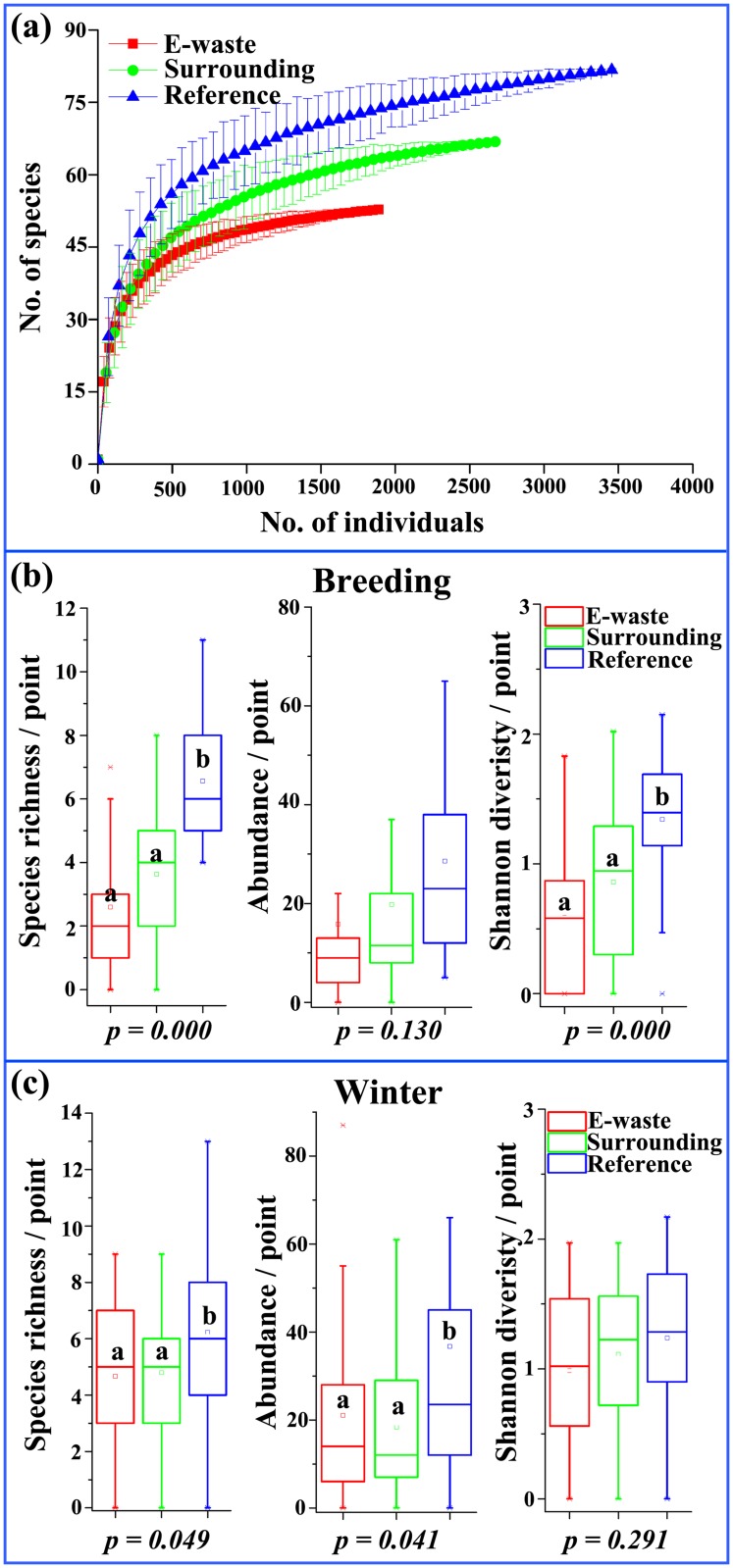
Sampled-based rarefaction curves (a); box and whisker plots comparing mean point species richness, abundance, and diversity among sites from e-waste exposed (*n = 30*), surrounding (*n = 30*) and reference sites (*n = 30*), occurring in breeding (b) and winter (c) season. Curves were generated by Monte-Carlo simulations with 95% confidence intervals (on the total number of species vs. individuals observed). Site estimates are based on standardized point counts where bird numbers were counted for 10 min at each plot. Each point was counted four times totally. Different letters at each box indicate significant differences at a = 0.05. *P* values are derived from *F* tests that use the type III sums-of-squares obtained from linear mixed models.

A total of 8,216 individuals from 104 species were recorded during the four surveys of the totaling 90 points (S1 Table). We observed 53, 67 and 82 bird species, and 1946, 2737 and 3538 individuals, in the e-waste exposed, surrounding and reference sites, respectively. Sampling saturation was achieved for all sites, as indicated by rapid approach of species number to asymptote (Fig. 2a). For the number of species and diversity (*H’*) per sampling point in breeding season, both of which were significantly affected by e-waste severity (exposed < surrounding < reference; species *F*_*2*,*6*_ = 30.2, *p* < 0.001; diversity *F*_*2*,*6*_ = 13.8, *p* < 0.001), and the variation was less obvious between the e-waste exposed and surrounding zone (post hoc, *p* = 0.159) (Fig. 2b). However, diversity was not different in winter (*F*_*2*,*6*_ = 1.3, *p = 0*.*291*) (Fig. 2c). Overall, the relative abundance and diversity were lower in, and decreased towards, the vicinity of the pollution source.

### Species similarity between sampling units (beta diversity)

**Fig 3:**
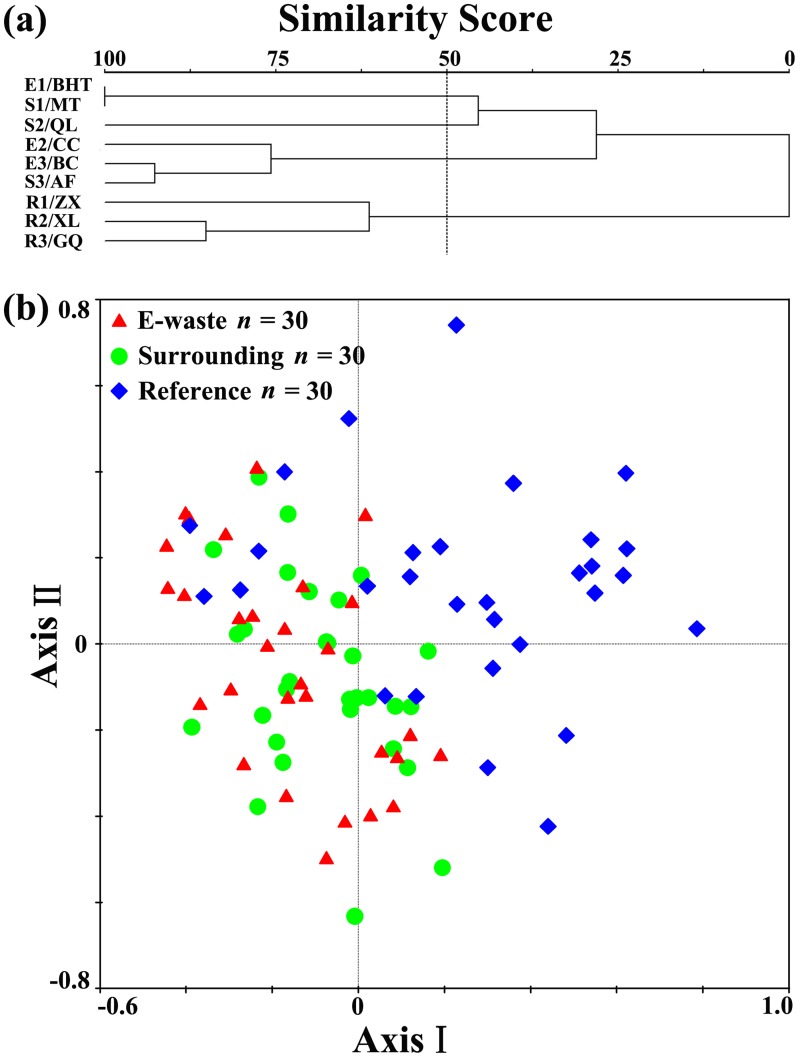
(a) Cluster analysis of bird communities of the 9 transects-villages using Sorensen similarity index as input. (b) Correspondence analysis (CA) ordination of count points with superimposed groups from e-waste exposed (*n = 30*), surrounding (*n = 30*) and reference sites (*n = 30*). See Fig. 1 for map of sites, and S1 Table for a summary of bird abundances.

The dissimilarities within e-waste exposed and natural farmlands were accentuated in community composition. A cluster analysis of bird community profiles at nine transects across the entire sampling area yielded two major groups, with two minor within-clusters mixed in the e-waste area. At the e-waste group, pairwise similarity of bird species composition was high between e-waste exposed and surrounding sites (0.75 ± 0.04) (Fig. 3a). But it was low between e-waste exposed (0.51 ± 0.09) or surrounding (0.46 ± 0.08) and reference sites. CA ordination also revealed site-specific patterns at the 90 count points, reflecting a community difference between e-waste polluted and natural farmlands. A graphical overlay on the ordination clearly distinguished points in the e-waste region (including both exposed and surrounding sites) from those natural farmlands, and the e-waste exposed mingled closely with surrounding sites while natural farmland sites clustered in the right quadrants (Fig. 3b). As a result, we interpret these clusters as two distinct bird community types, because of the relative proportions of the various guilds represented in the species that occur at the sites in each cluster (more details below).

### Assemblage of bird functional guilds

**Fig 4:**
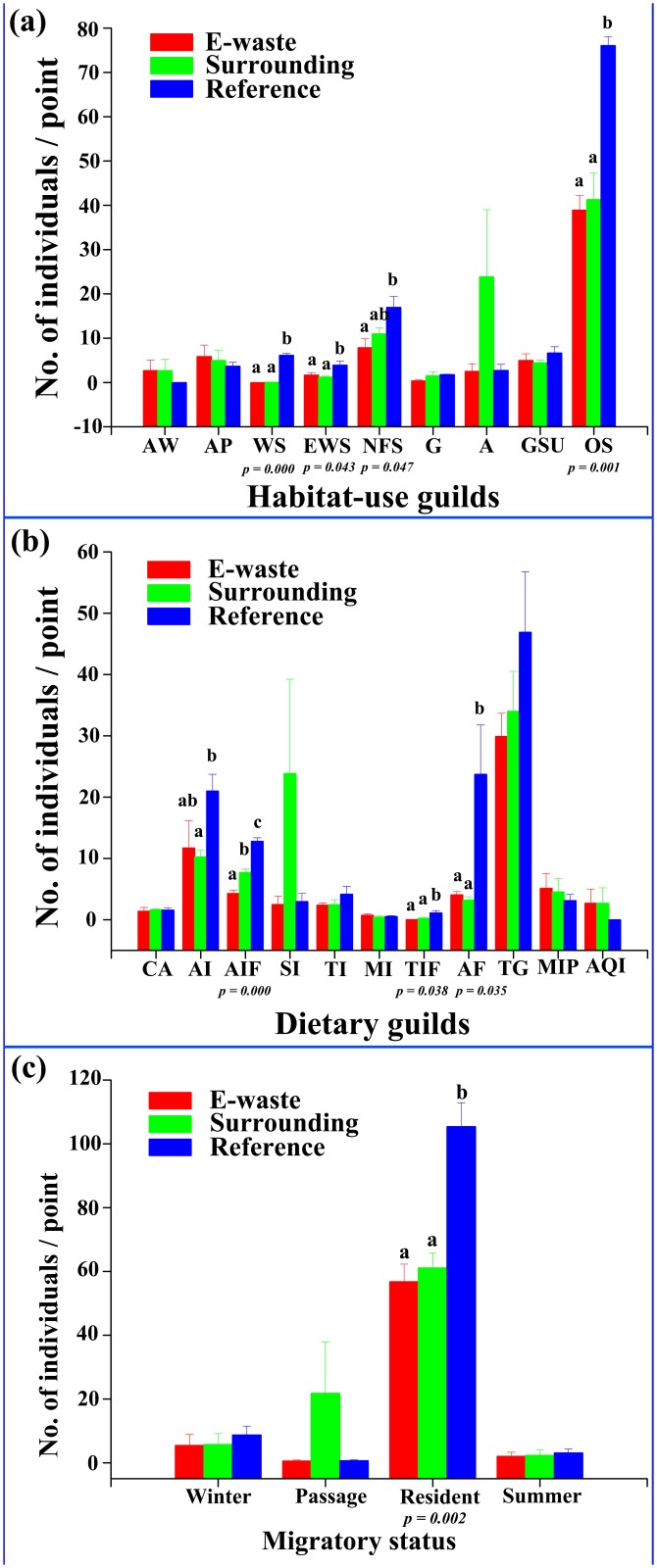
Changes in the relative abundance of birds grouped by (a) habitat preference, (b) dietary guilds, and (c) migratory status. Habitat preference codes: “artificial marshland or wetland (AW)”; “aquatic ponds and paddy (AP)”; “woodland specialist (WS)”; “edge-tolerant woodland species (EWS)”; “non-forest dependent species (e.g., plantation and orchard; NFS)”; “generalist (G)”; “aerial species (A)”; “grassland and shrub users (GSU)” and “open-habitat species (OS)”. Dietary guild codes: “carnivore (CA)”, “arboreal foliage glean insectivore (AI)”, “arboreal foliage glean insectivore-frugivore (AIF)”, “sallying insectivore (SI)”, “terrestrial insectivore (TI)”, “miscellaneous insectivore (MI)”, “terrestrial insectivore-frugivore (TIF)”, “arboreal frugivore (AF)”, “terrestrial granivores (TG)”, “miscellaneous insectivore-piscivore (MIP)”, and “aquatic invertebrate (AQI)”. Different letters at each box indicate significant differences at a = 0.05. *P* values are derived from *F* tests that use the type III sums-of-squares obtained from linear mixed models.

In terms of relative abundance of habitat-use guilds (Fig. 4a), open farmland birds formed the dominant guild, accounting for 57.1% of individuals counted at all points. Except for aquatic and aerial species (e.g., AW, AP and A), steady declines in relative abundance towards the pollution source appeared in woodland species (*F*_*2*,*6*_ = 165.0, *p* < 0.001), edge-tolerant forest species (*F*_*2*,*6*_ = 5.5, *p* = 0.043) and open farmland species (*F*_*2*,*6*_ = 24.8, *p* = 0.001), which had a significantly lower abundance in e-waste regions than those in natural reference farmlands. In fact, woodland species were hardly recorded in the e-waste exposed and surrounding areas.

Comparing dietary guilds, a pattern of decreasing bird abundance was also evident (Fig. 4b). Regarding open farmland species, all sites were dominated by granivores (mainly sparrows, munias, finches and buntings), which accounted for 40.5% of the total observed individuals. Moreover, there were more evenly distributed feeding guilds in natural farmland than in the e-waste region, and a marked decrease was found in e-waste sites that began with a lower proportion of arboreal foliage glean insectivores (*F*_*2*,*6*_ = 5.2, *p* = 0.046), arboreal foliage glean insectivore-frugivores (*F*_*2*,*6*_ = 58.7, *p* < 0.001), terrestrial insectivore-frugivores (*F*_*2*,*6*_ = 5.9, *p* = 0.038) and arboreal frugivores (*F*_*2*,*6*_ = 6.2, *p* = 0.035).

Of the 104 species with different migratory status (Fig. 4c), resident species composed of 81.4% of total individuals, whereas migrants and passages made up only 18.6%. None but resident species in relative abundance was higher on natural farmland than e-waste polluted zone (*F*_*2*,*6*_ = 20.2, *p* = 0.002). As noted by seasonal changes, since diversity were not different in winters (Fig. 2b, 2c), it stressed that migrants population were less impacted by e-waste severity.

### Species-specific and guild-dependent responses

When functional guilds were analyzed by species-level ordination, the distribution patterns of birds highlighted a more specific habitat preference. For instance, the species that decreased in the polluted areas mainly included arboreal insectivores or frugivores, and grassland insectivorous specialists, especially for WS (e.g., understory babblers, canopy cuckoos and treepies), EWS (e.g., large mid-story drongos, flycatchers), NFS (e.g., bulbuls, white-eyes) and GSU (e.g., pheasants, grassbirds, and cisticolas). Notably, some open farmland generalists also seldom occurred in the e-waste polluted sites, such as arboreal frugivores (e.g., starlings, mynas), and terrestrial granivores (e.g., buntings, finches). Conversely, certain groups were more abundant in the e-waste polluted sites relative to the natural farmlands, as miscellaneous AP (e.g., egrets, bitterns, and plovers), and granivorous OS (e.g., sparrows).

Out of the 104 species recorded, 25 breeding species exclusively used natural farmland while three species occurred only on e-waste polluted zone (S1 Table). By simply asking whether species were affected by e-waste severity, nine species (Spotted Dove *Streptopelia chinensis*, Red-whiskered Bulbul *Pycnonotus jocosus*, Chinese Bulbul *P*. *sinensis*, Crested Myna *Acridotheres cristatellus*, Hwamei *Garrulax canorus*, Rufous-necked Scimitar Babbler *Pomatorhinus ruficollis*, Rufous-capped Babbler *Stachyris ruficeps*, Great Tit *Parus major* and Grey-capped Greenfinch *Chloris sinica*) that were detected frequently enough to include in a statistical analysis decreased significantly in relative abundance from reference to e-waste polluted points (Table 2).

**Table 2:** Relative abundance for nine resident species that were detected significantly decreased from natural reference to e-waste polluted zone.

Family	Species	Exposed	Surrounding	Reference	*F*	*p*
Columbidae	Spotted Dove	8.7 ± 1.9	8.7 ± 1.2	21.7 ± 4.3	7.141	**0.026**
Pycnonotidae	Red-whiskered Bulbul	4.3 ± 2.6	12.7 ± 5.5	32.3 ± 6.9	7.239	**0.025**
	Chinese Bulbul	37.3 ± 3.9	63.0 ± 7.0	72.0 ± 4.2	11.871	**0.008**
Sturnidae	Crested Myna	31.3± 11.2	12.7 ± 4.5	109.7 ± 15.6	20.481	**0.002**
Timaliidae	Hwamei	0	0	7.0 ± 2.0	10.253	**0.008**
	Rufous-necked Scimitar Babbler	0	0	17.3 ± 5.4	10.440	**0.011**
	Rufous-capped Babbler	0	0	5.3 ± 1.7	10.240	**0.012**
Paridae	Great Tit	3.3 ± 1.8	8.7 ± 3.3	14.7 ± 1.5	6.028	**0.037**
Fringillidae	Grey-capped Greenfinch	18.0 ± 11.4	14.7 ± 10.1	112.0 ± 25.7	10.280	**0.012**

*Note*: Species include only those with ≥ 4 detections and normal distributions. Relative abundance is expressed as mean number of birds detected within 50 m per point at each site. Results of *F* test, as well as table-wide significance (*p*) are also given. The *F* test of significance uses the type III sums-of-squares obtained from a linear mixed model.

## Discussion

### Change in overall bird species richness

Rather than a dichotomy in e-waste versus natural farmlands, we found specific bird assemblages that reflected clear functional differences among the various sites. The e-waste severity showed negative effects on total bird species richness, density, and diversity patterns in a decreasing order of impact as follows: exposed < surrounding < natural, thus following the order based on the exposure levels and distance from pollution sources. Few studies have directly compared the bird fauna in polluted farmland with other habitats. For example, one study in Russia and Finland, dealing with the impacts of non-ferrous smelters on bird population densities around four smelters, revealed a marked decrease in species diversity towards the pollution source [10]. In the UK, indirect effects of pesticides operating through the food chain have been proposed as a possible causal factor in the decline of farmland bird species, especially the Grey Partridge *Perdix perdix*, Corn Bunting *Miliaria calandra* and Yellowhammer *Emberiza citrinella* [7].

The species assembly in exposed and surrounding sites was more similar to each other than they were to that of natural areas, based on the declining and vanishing species/category. Recent evidence suggests that the level of PCBs and PBDEs at the exposed and surrounding sites are similar in the Qingyuan region [17,26,27], which reinforces the negative effect extending farther from the point sources of e-waste pollution. And surrounding sites may be following a similar pattern of guild loss to that experienced by exposed area, assuming that present-day declines accurately predict future extirpations in neighboring district. Evidence has shown that unsafe recycling procedures impact further regions via various transfer pathways such as riverine runoff, air and dust transport, or exportation of contaminated fish [14,16].

### Disentangling the differential responses in species and guilds level

#### Woodland insectivores

Our study showed that species richness of woodland insectivores decreased in e-waste exposed and surrounding sites compared with natural farmland, which was most apparent in the arboreal gleaners and terrestrial insectivores. The former contained five species of cuckoos, two species of treepies, one woodpecker, and one long-tailed tit. The latter was composed of five babblers. Insectivores have been considered especially vulnerable to forest modification and seem to avoid the forest edge [37, 38]. Two possible explanations for this negative result are food scarcity [39], and fragmentation-nest success [40].

Agroecosystems usually support a considerable bird diversity in the tropics [41, 42]. In South China, most natural farmlands are accompanied with fragmented woods, which usually have a complex vegetation structure and an increased variability in foraging substrates. Arboreal and terrestrial insectivores are vulnerable to habitat degradation, through which e-waste pollutants may affect the suitable habitat for breeding. Decreased breeding success has been documented for several breeding insectivorous birds around three smelters in Russia and Finland [9,10]. They also found that small passerines need extra calcium during their breeding, and the lack of calcium-rich food, such as snails, has resulted in inferior breeding success in acidified or metal polluted areas. Indeed, intense pollution exposure in our study area has providing evidence of PBDEs biomagnification from insects to frogs, with lower arthropod abundance [18]. So it is possible that the availability of suitable invertebrate food is reduced in the e-waste polluted areas.

#### Shrub and grassland specialist

There was a significant pattern of decreasing species richness with increasing e-waste severity in terrestrial and grassland specialists, and most of these species were insectivores. The terrestrial insectivore-frugivore group was composed of two partridges and one pheasant, while the shrub and grassland group contained a cistocola, a grassbird, and a laughingthrush. These species were exclusively recorded in the natural farmland where all species search for food by pouncing on arthropods from the ground, or gleaning them from shrubs and grasses. For instance, the Chinese Grassbird *Graminicola striatus* is currently treated as near threatened, as it is thought to be suffering substantial long-term habitat losses due to drainage, and severe degradation of grassland habitats [43].

Ruderal vegetation, rough grassland and scrub could also provide potential supplementary resources to those available within the productive areas of farmland birds. For grassland specialists, extensive effluent; through melting or stripping of chips by acid leaching this has probably been the main driver of the bird population in the e-waste recycling region. In the UK, field drainage and more intensive grassland management have reduced the availability of the insect food supply, while the conversion of former grassland to wasteland has also reduced the acreage of potential foraging habitat, especially for Eurasia Skylark *Alauda arvensis* and Meadow Pipit *Anthus pratensis* [6,44]. In our study, the generally missing of common grassland specialists (e.g. Oriental Skylark *A*. *gulgula* and Richard's Pipit *A*. *richardi*) in the e-waste region may be a result of higher pollutant drainage in the past, which has caused severe changes in microclimatic condition, and the subsequent food availability.

#### Open farmland granivores

This group of birds is dominated by sparrows, munias, finches and buntings, and showed a clear preference for natural farmlands. A high number of granivores could be regarded as an indication of traditional agriculture and less land-use impacts, which is maintaining a high diversity and richness of farmland birds. Although generally less specialized than insectivores, most granivores regularly use seed as food resources and supplement their diet with fruit on fruit trees to a varying extent. This preference could be attributed to the high availability of rice paddy resources in natural reference systems, as well as less pollution.

E-waste pollutants also affect paddy or weed seed production, thereby potentially reducing the availability of food resources for open farmland granivores. Earlier studies have successfully revealed that the use of herbicides and fertilizers depresses food supply in Europe for some seed-eaters, such as Yellowhammer [45], Linnet *Carduelis cannnabina* [46] and European Turtle Dove *Steptopelia turtur* [47] during the breeding season; Tree Sparrow *Passer montanus* [48], Reed Bunting *Emberiza schoeniclus* [49], European Greenfinch *C chloris* and European Goldfinch *Carduelis*. *carduelis* [50] in the non-breeding season. In our study areas, the emission of POPs may reduce current-year paddy seed production, and lead to long-term depletion of the seed bank in the soil.

#### Artificial wetland species

In contrast to terrestrial insectivores and granivores, most egrets and waders, which usually forage in water or wetland, seem to be tolerable to a wide range of e-waste contamination in exposed and surrounding areas. Although Luo et al. [17] has found that the concentrations of Persistent Halogenated Compounds (PHCs) in several waterbirds (e.g., Chinese Pond Heron *Ardeola bacchus*) in the e-waste recycling region were higher than those from most other previous studies with birds having a similar trophic level, there was no significant spatial trend in total bird density among the three habitats in the present study. This is likely due to a relatively higher activity of waders and egrets, and their home range could also be much larger than that of passerines. In addition, nearby pond culture offers birds more luxurious food resources, such as fish, weeds and small mollusks. We conclude that there is no evidence of contamination in aquatic bird populations and at a community level at the moment. Nerveless, more monitoring of birds residing at both e-waste and reference sites are necessary to confirm this conclusion in future.

### Conclusions and future research

The study has confirmed that improper e-waste dismantling activities result in a severe decline of bird functional assemblages around pollution sources, the effect of which appeared to be species-specific and guild-dependent. The species that decreased in the polluted areas were ecologically diverse, including both migratory and resident ones that are insectivorous and granivorous. Notably, the negatively affected species included those found almost exclusively in natural farmland (e.g., woodland insectivores and grassland specialists), or primarily in natural farmland, but also occurring in lower numbers in e-waste polluted zone (e.g., edge-tolerant species and open farmland specialists). By contrast, the response of waterbirds, omnivorous and non-breeding visitors is invariable so far. In addition, similar pattern of bird assembly between exposed and surrounding sites indicate that unsafe recycling procedures result in serious contamination clearly extending farther from the point sources of e-waste pollution.

Owning to the uncertainty of the exposure levels in a strictly dose-dependent manner, and how important indirect effects of e-waste POPs are in relation to other factors affecting bird population [e.g. life history traits and suitable resources (food and/or habitat)]. Further work is required in the following areas: (1) to investigate the impact of the results at different pollution levels, (2) to assess the impact of e-waste pollution, particularly on population-level responses of birds, and in a food-web context, (3) and to establish a long-term bird monitoring schemes at the same transects in e-waste polluted and reference sites.

## Supporting Information

S1 Table Ecological or functional categorizations and encounter rate (no of individuals per point) of all species recorded from e-waste exposed, surrounding, and reference sites in Guangdong, China. Data on three key ecological or functional traits (i.e. habitat preference, dietary guild and migratory status) were primarily collated from Zhao (2001) and Zhang et al. (2011). ^a^Habitat preference codes: “artificial marshland or wetland (AW)”; “aquatic ponds and paddy (AP)”; “woodland specialist (WS)”; “edge-tolerant woodland species (EWS)”; “non-forest dependent species (e.g., plantation and orchard; NFS)”; “generalist (G)”; “aerial species (A)”; “grassland and shrub users (GSU)” and “open-habitat species (OS)”. ^b^Dietary guild codes: “carnivore (CA)”, “arboreal foliage glean insectivore (AI)”, “arboreal foliage glean insectivore-frugivore (AIF)”, “sallying insectivore (SI)”, “terrestrial insectivore (TI)”, “miscellaneous insectivore (MI)”, “terrestrial insectivore-frugivore (TIF)”, “arboreal frugivore (AF)”, “terrestrial granivores (TG)”, “miscellaneous insectivore-piscivore (MIP)”, and “aquatic invertebrate (AQI)”. ^c^Migrations status codes: Permanent resident (R), Winter visitor (W), Summer visitor (S), and Passage migrant (P).
(DOC)
