# Supplementary material for: Do Bird Assemblages Predict Susceptibility by E-Waste Pollution? A Comparative Study Based on Species- and Guild-Dependent Responses in China Agroecosystems
Source: PLoS One. 2015 Mar 26;10(3):e0122264. doi: 10.1371/journal.pone.0122264 (PMC4374810; doi:10.1371/journal.pone.0122264)
Supplement: S1 Table — aHabitat preference codes: “artificial marshland or wetland (AW)”; “aquatic ponds and paddy (AP)”; “woodland specialist (WS)”; “edge-tolerant woodland species (EWS)”; “non-forest dependent species (e.g., plantation and orchard; NFS)”; “generalist (G)”; “aerial species (A)”; “grassland and shrub users (GSU)” and “open-habitat species (OS)”. bDietary guild codes: “carnivore (CA)”, “arboreal foliage glean insectivore (AI)”, “arboreal foliage glean insectivore-frugivore (AIF)”, “sallying insectivore (SI)”, “terrestrial insectivore (TI)”, “miscellaneous insectivore (MI)”, “terrestrial insectivore-frugivore (TIF)”, “arboreal frugivore (AF)”, “terrestrial granivores (TG)”, “miscellaneous insectivore-piscivore (MIP)”, and “aquatic invertebrate (AQI)”. cMigrations status codes: Permanent resident (R), Winter visitor (W), Summer visitor (S), and Passage migrant (P). (DOC) [file pone.0122264.s001.doc]

**Table S1. Ecological or functional categorizations and encounter rate (no of individuals per point) of all species recorded from e-waste exposed, surrounding, and reference sites in Guangdong, China. Data on three key ecological or functional traits (i.e. habitat preference, dietary guild and migratory status) were primarily collated from Zhao (2001) and Zhang et al. (2011).**

^a^ Habitat preference codes: “artificial marshland or wetland (AW)”; “aquatic ponds and paddy (AP)”; “woodland specialist (WS)”; “edge-tolerant woodland species (EWS)”; “non-forest dependent species (e.g., plantation and orchard; NFS)”; “generalist (G)”; “aerial species (A)”; “grassland and shrub users (GSU)” and “open-habitat species (OS)”.

^b^ Dietary guild codes: “carnivore (CA)”, “arboreal foliage glean insectivore (AI)”, “arboreal foliage glean insectivore-frugivore (AIF)”, “sallying insectivore (SI)”, “terrestrial insectivore (TI)”, “miscellaneous insectivore (MI)”, “terrestrial insectivore-frugivore (TIF)”, “arboreal frugivore (AF)”, “terrestrial granivores (TG)”, “miscellaneous insectivore-piscivore (MIP)”, and “aquatic invertebrate (AQI)”.

^c^ Migrations status codes: Permanent resident (R), Winter visitor (W), Summer visitor (S), and Passage migrant (P).

| **Common name** | **Scientific name** | **Habitat^a^** | **Diet^b^** | **Status^c^** | **Exposed** | | | **Surrounding** | | | **Reference** | | |
| --- | --- | --- | --- | --- | --- | --- | --- | --- | --- | --- | --- | --- | --- |
|  |  |  |  |  | **BHT**  ***n* =10** | **CC**  ***n* =10** | **BC**  ***n* =10** | **MT**  ***n* =10** | **QL**  ***n* =10** | **AF**  ***n* =10** | **ZX**  ***n* =10** | **XL**  ***n* =10** | **GQ**  ***n* =10** |
| Little Grebe | *Tachybapus ruficollis* | AP | MIP | R | 0.30 | 0.23 | 0.13 | 0.23 | 0.00 | 0.05 | 0.05 | 0.05 | 0.00 |
| Grey Heron | *Ardea cinerea* | AP | MIP | W | 0.00 | 0.00 | 0.00 | 0.05 | 0.00 | 0.00 | 0.00 | 0.00 | 0.00 |
| Great Egret | *Ardea alba* | AP | MIP | W | 0.00 | 0.00 | 0.00 | 0.00 | 0.00 | 0.08 | 0.00 | 0.00 | 0.00 |
| Intermediate Egret | *Egretta intermedia* | AP | MIP | W | 0.00 | 0.00 | 0.03 | 0.00 | 0.00 | 0.00 | 0.00 | 0.00 | 0.00 |
| Little Egret | *Egretta garzetta* | AP | MIP | R | 0.50 | 0.03 | 0.05 | 0.53 | 0.00 | 0.23 | 0.35 | 0.08 | 0.45 |
| Cattle Egret | *Bubulcus ibis* | AP | MIP | R | 1.13 | 0.00 | 0.00 | 0.83 | 0.00 | 0.00 | 0.33 | 0.00 | 0.20 |
| Chinese Pond Heron | *Ardeola bacchus* | AP | MIP | R | 0.33 | 0.10 | 0.00 | 0.23 | 0.05 | 0.20 | 0.20 | 0.08 | 0.33 |
| Green-backed Heron | *Butorides striatus* | AP | MIP | R | 0.00 | 0.05 | 0.10 | 0.00 | 0.00 | 0.05 | 0.00 | 0.00 | 0.00 |
| Black-crowned Night Heron | *Nycticorax nycticorax* | AP | MIP | P | 0.00 | 0.00 | 0.00 | 0.00 | 0.00 | 0.00 | 0.03 | 0.00 | 0.00 |
| Chinese Little Bittern | *Ixobrychus sinensis* | AP | MIP | R | 0.15 | 0.30 | 0.00 | 0.10 | 0.10 | 0.05 | 0.00 | 0.00 | 0.03 |
| Cinnamon Bittern | *Ixobrychus cinnamomeus* | AP | MIP | R | 0.00 | 0.00 | 0.15 | 0.03 | 0.05 | 0.05 | 0.03 | 0.03 | 0.00 |
| Black-shouldered Kite | *Elanus caeruleus* | A | CA | P | 0.10 | 0.00 | 0.00 | 0.13 | 0.00 | 0.03 | 0.03 | 0.03 | 0.00 |
| Common Buzzard | *Buteo buteo* | G | CA | W | 0.00 | 0.03 | 0.00 | 0.00 | 0.00 | 0.03 | 0.00 | 0.00 | 0.00 |
| Common Kestrel | *Falco tinnunculus* | A | CA | W | 0.10 | 0.00 | 0.00 | 0.00 | 0.08 | 0.05 | 0.00 | 0.03 | 0.00 |
| Common Pheasant | *Phasianus colchicus* | GSU | TIF | R | 0.00 | 0.00 | 0.00 | 0.00 | 0.00 | 0.00 | 0.00 | 0.30 | 0.00 |
| Japanese Quail | *Coturnix japonica* | GSU | TIF | W | 0.00 | 0.00 | 0.00 | 0.00 | 0.00 | 0.00 | 0.03 | 0.00 | 0.00 |
| Chinese Bamboo Partridge | *Bambusicola thoracicus* | GSU | TIF | R | 0.00 | 0.00 | 0.00 | 0.00 | 0.00 | 0.00 | 0.03 | 0.00 | 0.03 |
| Slaty-breasted Rail | *Gallirallus striatus* | AP | MI | R | 0.03 | 0.00 | 0.03 | 0.00 | 0.00 | 0.05 | 0.00 | 0.08 | 0.03 |
| White-breasted Waterhen | *Amaurornis phoenicurus* | AP | MI | R | 0.13 | 0.05 | 0.20 | 0.03 | 0.05 | 0.15 | 0.08 | 0.13 | 0.08 |
| Common Moorhen | *Gallinula chloropus* | AP | MI | R | 0.13 | 0.00 | 0.00 | 0.05 | 0.00 | 0.00 | 0.03 | 0.00 | 0.00 |
| Little Ringed Plover | *Charadrius dubius* | AW | AQI | W | 0.00 | 0.00 | 0.00 | 0.00 | 0.00 | 0.58 | 0.00 | 0.00 | 0.00 |
| Kentish Plover | *Charadrius alexandrinus* | AW | AQI | W | 0.00 | 0.00 | 0.00 | 0.00 | 0.00 | 0.13 | 0.00 | 0.00 | 0.00 |
| Pintail Snipe | *Gallinago stenura* | AW | AQI | W | 0.00 | 0.00 | 0.00 | 0.03 | 0.00 | 0.00 | 0.00 | 0.00 | 0.00 |
| Spotted Redshank | *Tringa erythropus* | AW | AQI | W | 0.00 | 0.00 | 0.00 | 0.00 | 0.00 | 0.50 | 0.00 | 0.00 | 0.00 |
| Common Greenshank | *Tringa nebularia* | AW | AQI | W | 0.00 | 0.00 | 0.00 | 0.00 | 0.00 | 0.25 | 0.00 | 0.00 | 0.00 |
| Green Sandpiper | *Tringa ochropus* | AW | AQI | W | 0.08 | 0.03 | 0.05 | 0.00 | 0.00 | 0.03 | 0.00 | 0.00 | 0.00 |
| Wood Sandpiper | *Tringa glareola* | AW | AQI | W | 1.75 | 0.13 | 0.00 | 0.05 | 0.00 | 0.00 | 0.00 | 0.00 | 0.00 |
| Common Sandpiper | *Actitis hypoleucos* | AW | AQI | W | 0.00 | 0.00 | 0.00 | 0.03 | 0.00 | 0.45 | 0.00 | 0.00 | 0.00 |
| Oriental Turtle Dove | *Streptopelia orientalis* | G | TG | R | 0.00 | 0.00 | 0.00 | 0.00 | 0.38 | 0.00 | 0.00 | 0.05 | 0.03 |
| Spotted Dove | *Streptopelia chinensis* | OS | TG | R | 0.28 | 0.25 | 0.13 | 0.18 | 0.20 | 0.28 | 0.65 | 0.65 | 0.33 |
| Large Hawk-Cuckoo | *Hierococcyx sparverioides* | FS | AI | S | 0.00 | 0.00 | 0.00 | 0.00 | 0.00 | 0.00 | 0.05 | 0.00 | 0.05 |
| Indian Cuckoo | *Cuculus micropterus* | FS | AI | S | 0.00 | 0.00 | 0.00 | 0.00 | 0.00 | 0.00 | 0.03 | 0.00 | 0.00 |
| Asian Lesser Cuckoo | *Cuculus poliocephalus* | EWS | AI | S | 0.00 | 0.00 | 0.00 | 0.00 | 0.00 | 0.00 | 0.03 | 0.00 | 0.03 |
| Plaintive Cuckoo | *Cacomantis merulinus* | G | AI | S | 0.00 | 0.00 | 0.00 | 0.03 | 0.05 | 0.00 | 0.05 | 0.00 | 0.03 |
| Fork-tailed Drongo-Cuckoo | *Surniculus dicruroides* | EWS | SI | S | 0.00 | 0.00 | 0.00 | 0.00 | 0.00 | 0.00 | 0.03 | 0.00 | 0.03 |
| Asian Koel | *Eudynamys scolopaceus* | FS | AI | S | 0.00 | 0.00 | 0.00 | 0.00 | 0.00 | 0.05 | 0.00 | 0.10 | 0.10 |
| Common Coucal | *Centropus sinensis* | GSU | TI | R | 0.05 | 0.15 | 0.00 | 0.05 | 0.08 | 0.13 | 0.10 | 0.13 | 0.25 |
| Lesser Coucal | *Centropus bengalensis* | GSU | TI | R | 0.00 | 0.00 | 0.00 | 0.03 | 0.00 | 0.08 | 0.00 | 0.10 | 0.10 |
| Common Kingfisher | *Alcedio atthis* | AP | MIP | R | 0.08 | 0.10 | 0.10 | 0.15 | 0.10 | 0.18 | 0.03 | 0.05 | 0.03 |
| White-throated Kingfisher | *Halcyon smyrnensis* | AP | MIP | R | 0.00 | 0.00 | 0.00 | 0.00 | 0.00 | 0.05 | 0.00 | 0.00 | 0.00 |
| Black-caped Kingfisher | *Halcyon pileata* | AP | MIP | R | 0.00 | 0.03 | 0.00 | 0.00 | 0.00 | 0.00 | 0.00 | 0.00 | 0.00 |
| Lesser Pied Kingfisher | *Ceryle rudis* | AP | MIP | R | 0.00 | 0.00 | 0.00 | 0.05 | 0.00 | 0.00 | 0.03 | 0.00 | 0.03 |
| Wryneck | *Jynx torquilla* | EWS | AI | W | 0.00 | 0.00 | 0.00 | 0.00 | 0.00 | 0.00 | 0.00 | 0.03 | 0.00 |
| Oriental Skylark | *Alauda gulgula* | OS | TG | W | 0.00 | 0.00 | 0.00 | 0.00 | 0.00 | 0.03 | 0.00 | 0.00 | 0.00 |
| Barn Swallow | *Hirundo rustica* | A | SI | S | 1.13 | 0.00 | 0.25 | 1.38 | 0.00 | 0.05 | 0.13 | 0.38 | 1.10 |
| Red-rumped Swallow | *Hirundo daurica* | A | SI | P | 0.13 | 0.00 | 0.20 | 1.45 | 13.43 | 1.33 | 0.13 | 0.00 | 0.25 |
| White Wagtail | *Motacilla alba* | OS | TI | R | 0.33 | 0.30 | 0.48 | 0.25 | 0.30 | 0.38 | 0.28 | 0.20 | 0.30 |
| Yellow Wagtail | *Motacilla flava* | OS | TI | W | 0.00 | 0.05 | 0.00 | 0.00 | 0.00 | 0.00 | 0.00 | 0.00 | 0.00 |
| Richard's Pipit | *Anthus richardi* | OS | TI | W | 0.20 | 0.00 | 0.00 | 0.00 | 0.08 | 0.33 | 0.20 | 0.13 | 0.05 |
| Olive-backed Pipit | *Anthus hodgsoni* | NFS | TI | W | 0.00 | 0.00 | 0.00 | 0.00 | 0.08 | 0.08 | 0.05 | 0.20 | 0.13 |
| Ashy Minivet | *Pericrocotus divaricatus* | EWS | AI | P | 0.00 | 0.00 | 0.03 | 0.00 | 0.00 | 0.00 | 0.00 | 0.00 | 0.00 |
| Collared Finchbill | *Spizixos semitorques* | NFS | AIF | R | 0.00 | 0.00 | 0.00 | 0.00 | 0.00 | 0.00 | 0.03 | 0.05 | 0.18 |
| Red-whiskered Bulbul | *Pycnonotus jocosus* | NFS | AIF | R | 0.00 | 0.23 | 0.10 | 0.28 | 0.58 | 0.10 | 1.10 | 0.50 | 0.83 |
| Chinese Bulbul | *Pycnonotus sinensis* | NFS | AIF | R | 0.88 | 1.13 | 0.80 | 1.40 | 1.40 | 1.93 | 1.75 | 1.65 | 2.00 |
| Sooty-headed Bulbul | *Pycnonotus aurigaster* | NFS | AIF | R | 0.00 | 0.00 | 0.10 | 0.00 | 0.00 | 0.08 | 0.15 | 0.43 | 0.13 |
| Chestnut Bulbul | *Hemixos castanonotus* | FS | AIF | R | 0.00 | 0.00 | 0.00 | 0.00 | 0.00 | 0.00 | 0.05 | 0.00 | 0.05 |
| Long-tailed Shrike | *Lanius schach* | OS | CA | R | 0.45 | 0.15 | 0.23 | 0.30 | 0.38 | 0.25 | 0.23 | 0.55 | 0.33 |
| Black Drongo | *Dicrurus macrocercus* | EWS | SI | S | 0.03 | 0.13 | 0.00 | 0.03 | 0.13 | 0.05 | 0.05 | 0.13 | 0.03 |
| Crested Myna | *Acridotheres cristatellus* | OS | AF | R | 0.23 | 1.08 | 1.05 | 0.10 | 0.48 | 0.38 | 2.08 | 3.43 | 2.73 |
| Black-collared Starling | *Gracupica nigricollis* | OS | AF | R | 0.55 | 0.15 | 0.00 | 0.90 | 0.05 | 0.50 | 0.13 | 0.53 | 0.25 |
| White-shouldered Starling | *Sturnia sinensis* | OS | AF | R | 0.00 | 0.00 | 0.00 | 0.00 | 0.00 | 0.00 | 7.60 | 0.00 | 0.00 |
| Silky Starling | *Sturnus sericeus* | OS | AF | W | 0.00 | 0.00 | 0.00 | 0.00 | 0.00 | 0.00 | 0.00 | 1.00 | 0.08 |
| Eurasian Jay | *Garrulus glandarius* | FS | AIF | R | 0.00 | 0.00 | 0.00 | 0.00 | 0.00 | 0.00 | 0.00 | 0.03 | 0.00 |
| Red-billed Blue Magpie | *Urocissa erythrorhyncha* | EWS | AIF | R | 0.00 | 0.00 | 0.00 | 0.00 | 0.00 | 0.00 | 0.00 | 0.23 | 0.20 |
| Gray Treepie | *Dendrocitta formosae* | FS | AIF | R | 0.00 | 0.00 | 0.00 | 0.00 | 0.00 | 0.00 | 0.03 | 0.00 | 0.00 |
| Common Magpie | *Pica pica* | OS | AIF | R | 0.00 | 0.00 | 0.00 | 0.00 | 0.03 | 0.03 | 0.18 | 0.00 | 0.00 |
| Large-billed Crow | *Corvus macrorhynchos* | G | AIF | R | 0.00 | 0.00 | 0.00 | 0.00 | 0.00 | 0.00 | 0.00 | 0.05 | 0.03 |
| Magpie Robin | *Copsychus saularis* | OS | AI | R | 0.40 | 0.50 | 0.23 | 0.28 | 0.30 | 0.33 | 0.23 | 0.30 | 0.40 |
| Daurian Redstart | *Phoenicurus auroreus* | OS | AI | W | 0.00 | 0.03 | 0.03 | 0.00 | 0.00 | 0.05 | 0.08 | 0.08 | 0.10 |
| Stonechat | *Saxicola torquata* | GSU | AI | W | 0.28 | 0.13 | 0.00 | 0.28 | 0.10 | 0.28 | 0.08 | 0.13 | 0.10 |
| Blue Whistling Thrush | *Myiophonus caeruleus* | EWS | TIF | S | 0.00 | 0.00 | 0.00 | 0.00 | 0.00 | 0.00 | 0.00 | 0.00 | 0.03 |
| Orange-headed Thrush | *Zoothera citrina* | FS | TIF | P | 0.00 | 0.00 | 0.00 | 0.00 | 0.00 | 0.00 | 0.05 | 0.00 | 0.00 |
| Blackbird | *Turdus merula* | NFS | TIF | R | 0.03 | 0.00 | 0.00 | 0.03 | 0.15 | 0.00 | 0.00 | 0.10 | 0.30 |
| Sooty Flycatcher | *Muscicapa sibirica* | EWS | SI | W | 0.00 | 0.00 | 0.03 | 0.00 | 0.05 | 0.00 | 0.00 | 0.00 | 0.00 |
| Asian Brown Flycatcher | *Muscicapa dauurica* | EWS | SI | W | 0.00 | 0.00 | 0.00 | 0.03 | 0.00 | 0.00 | 0.00 | 0.00 | 0.00 |
| Spectacled Laughingthrush | *Garrulax perspicillatus* | GSU | TI | R | 0.00 | 0.25 | 0.00 | 0.00 | 0.00 | 0.03 | 0.00 | 0.40 | 0.00 |
| Hwamei | *Garrulax canorus* | FS | AI | R | 0.00 | 0.00 | 0.00 | 0.00 | 0.00 | 0.00 | 0.28 | 0.13 | 0.13 |
| White-browed Laughingthrush | *Pterorhinus sannio* | GSU | TI | R | 0.00 | 0.00 | 0.00 | 0.00 | 0.00 | 0.00 | 0.00 | 0.53 | 0.00 |
| Rufous-necked Scimitar Babbler | *Pomatorhinus ruficollis* | FS | AI | R | 0.00 | 0.00 | 0.00 | 0.00 | 0.00 | 0.00 | 0.18 | 0.50 | 0.63 |
| Rusty-cheeked Scimitar Babbler | *Pomatorhinus erythrogenys* | FS | AI | R | 0.00 | 0.00 | 0.00 | 0.00 | 0.00 | 0.00 | 0.03 | 0.00 | 0.00 |
| Rufous-caped Babbler | *Stachyris ruficeps* | FS | AI | R | 0.00 | 0.00 | 0.00 | 0.00 | 0.00 | 0.00 | 0.05 | 0.18 | 0.18 |
| Grey-cheeked Fulvetta | *Alcippe morrisonia* | FS | AI | R | 0.00 | 0.00 | 0.00 | 0.00 | 0.00 | 0.00 | 0.20 | 0.00 | 0.50 |
| Zitting Cisticola | *Cisticola juncidis* | GSU | AI | R | 0.00 | 0.00 | 0.00 | 0.00 | 0.00 | 0.00 | 0.00 | 0.03 | 0.00 |
| Yellow-bellied Prinia | *Prinia flaviventris* | GSU | AI | R | 0.80 | 0.23 | 0.43 | 0.60 | 0.33 | 0.58 | 0.53 | 0.43 | 0.50 |
| Plain Prinia | *Prinia inornata* | GSU | AI | R | 0.83 | 0.38 | 0.23 | 0.28 | 0.30 | 0.23 | 0.45 | 0.30 | 0.48 |
| Brownish-flanked Bush Warbler | *Cettia fortipes* | EWS | AI | R | 0.00 | 0.00 | 0.00 | 0.00 | 0.00 | 0.00 | 0.10 | 0.03 | 0.53 |
| Common Tailorbird | *Orthotomus sutorius* | OS | AI | R | 0.15 | 0.18 | 0.03 | 0.08 | 0.10 | 0.05 | 0.15 | 0.18 | 0.28 |
| Dusky Warbler | *Phylloscopus fuscatus* | EWS | AI | W | 0.18 | 0.03 | 0.03 | 0.05 | 0.08 | 0.13 | 0.20 | 0.15 | 0.13 |
| Pallas's Leaf Warbler | *Phylloscopus proregulus* | EWS | AI | W | 0.08 | 0.00 | 0.00 | 0.00 | 0.00 | 0.05 | 0.00 | 0.25 | 0.00 |
| Yellow-browed Warbler | *Phylloscopus inornatus* | EWS | AI | W | 0.38 | 0.30 | 0.08 | 0.10 | 0.10 | 0.15 | 0.15 | 0.43 | 0.23 |
| Chinese Grassbird | *Graminicola striatus* | GSU | AI | R | 0.00 | 0.00 | 0.00 | 0.00 | 0.00 | 0.00 | 0.00 | 0.03 | 0.00 |
| Japanese White-eye | *Zosterops japonicus* | NFS | AI | R | 1.75 | 0.90 | 0.00 | 1.00 | 1.15 | 0.00 | 1.25 | 0.20 | 1.68 |
| Red-headed Tit | *Aegithalos concinnus* | FS | AI | R | 0.00 | 0.00 | 0.00 | 0.00 | 0.00 | 0.00 | 0.75 | 0.38 | 0.00 |
| Great Tit | *Parus major* | G | AI | R | 0.10 | 0.15 | 0.00 | 0.10 | 0.38 | 0.18 | 0.38 | 0.30 | 0.43 |
| Tree Sparrow | *Passer montanus* | OS | TG | R | 5.25 | 1.50 | 4.55 | 4.63 | 8.58 | 4.35 | 1.70 | 0.13 | 3.05 |
| White-rumped Munia | *Lonchura striata* | OS | TG | R | 0.00 | 0.50 | 0.00 | 0.08 | 0.15 | 0.75 | 0.00 | 0.00 | 1.43 |
| Spotted Munia | *Lonchura punctulata* | OS | TG | R | 1.13 | 3.13 | 4.25 | 0.30 | 1.63 | 2.85 | 0.50 | 9.03 | 6.63 |
| Brambing | *Fringilla montifriingilla* | NFS | TG | P | 0.00 | 0.00 | 0.00 | 0.00 | 0.03 | 0.00 | 0.00 | 0.00 | 0.00 |
| Grey-capped Greenfinch | *Chloris sinica* | OS | TG | R | 0.98 | 0.38 | 0.00 | 0.25 | 0.00 | 0.85 | 2.30 | 2.03 | 4.08 |
| Black-tailed Grosbeak | *Eophona migratoria* | OS | TG | W | 0.05 | 0.00 | 0.00 | 0.00 | 0.03 | 0.00 | 0.00 | 0.00 | 0.00 |
| Crested Bunting | *Melophus lathami* | NFS | TG | R | 0.00 | 0.00 | 0.00 | 0.00 | 0.00 | 0.00 | 0.00 | 0.00 | 0.05 |
| Little Bunting | *Emberiza pusilla* | OS | TG | W | 0.00 | 0.00 | 0.00 | 0.00 | 0.00 | 0.00 | 1.80 | 0.38 | 0.00 |
| Yellow-browed Bunting | *Emberiza chrysophrys* | OS | TG | W | 0.00 | 0.00 | 0.00 | 0.00 | 0.00 | 0.00 | 0.00 | 0.08 | 0.00 |
| Black-faced Bunting | *Emberiza spodocephala* | OS | TG | W | 0.00 | 0.00 | 0.08 | 0.00 | 0.00 | 0.00 | 0.18 | 0.15 | 0.00 |
